# Genomic context of mutations in *MIR142* in blood cancers: summary and integrative analysis

**DOI:** 10.1186/s11658-026-00933-9

**Published:** 2026-05-18

**Authors:** Wladyslaw Wegorek, Adrian Tire, Daniel Kuznicki, Julia Richter, Maciej Giefing, Wolfram Klapper, Piotr Kozlowski, Paulina Galka-Marciniak

**Affiliations:** 1 Department of Molecular Genetics, Institute of Bioorganic Chemistry, Polish Academy of Sciences, Poznan, Poland; 2 Hematopathology Section, Department of Pathology, University Hospital Schleswig-Holstein, Kiel, Germany; 3 Institute of Human Genetics, Polish Academy of Sciences, Poznan, Poland

**Keywords:** miR-142, DLBCL, AML, Lymphoma, miRNA mutations, CLL

## Abstract

*MIR142* is the most frequently mutated microRNA (miRNA) gene in cancer, with recurrent alterations observed particularly in hematologic malignancies of lymphoid origin. It is expressed at very high levels in blood cells and plays an essential role in the development, differentiation, and maturation of various lymphopoietic and hematopoietic lineages. To gain a deeper understanding of *MIR142* mutations, we summarize all data on these mutations, including their frequency in different cancers, their location within the miR-142 precursor, and their functional consequences. We also analyzed *MIR142* mutations within a broader genomic context in thousands of cancers, including hundreds of blood neoplasms. Our results show that the most prevalent mutations in the *MIR142* gene originate from a clearly distinct hotspot in hematologic malignancies, concentrated mainly within the sequence of the secondary miR-142 precursor. We found substantial differences in mutation frequency and distribution across cancer types. This indicates that *MIR142* alterations are not random consequences of an increased mutational load but are likely subject to positive selection, underscoring their biological and clinical significance.

**Graphical Abstract:**

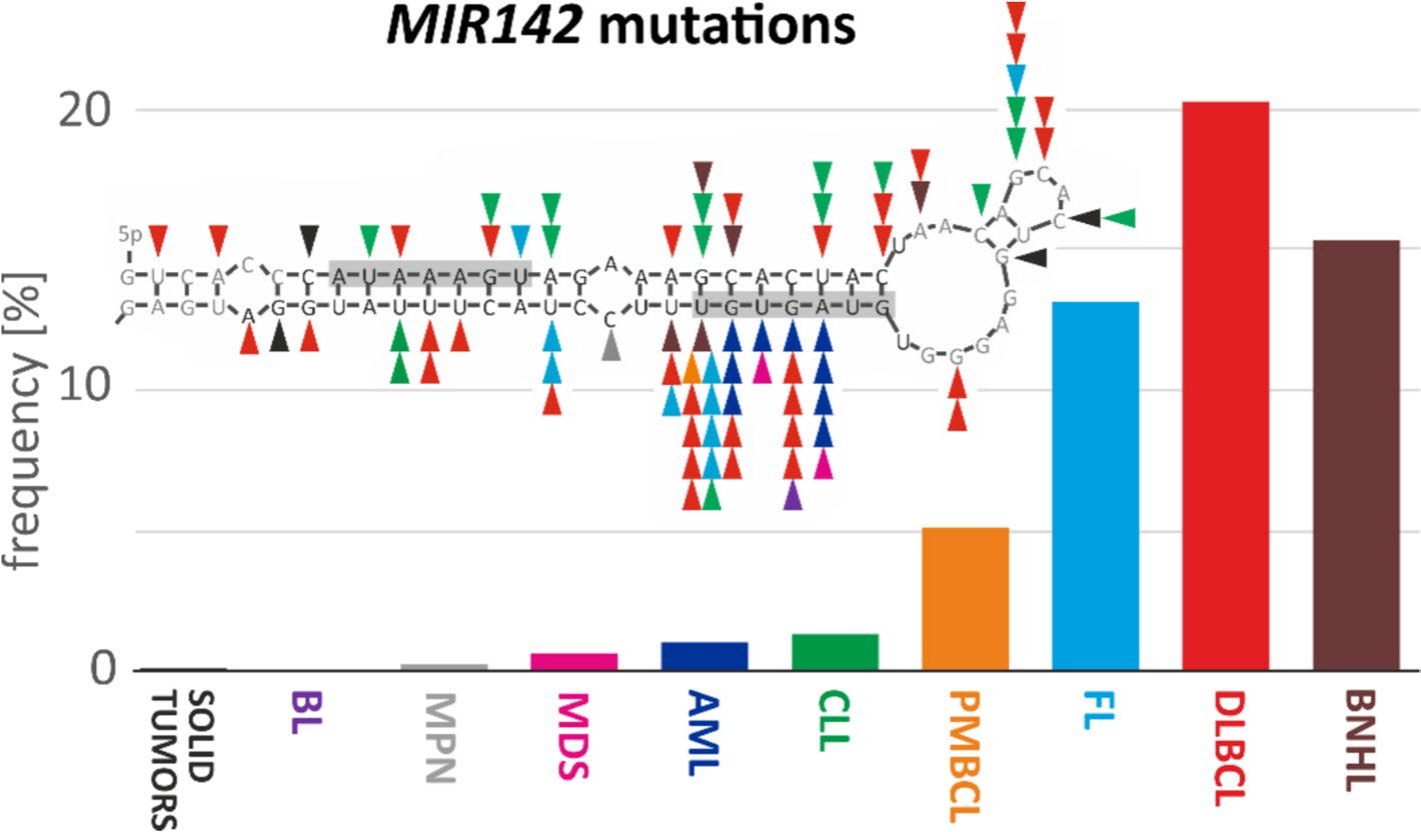

**Supplementary Information:**

The online version contains supplementary material available at 10.1186/s11658-026-00933-9.

## Introduction

**Fig. 1:**
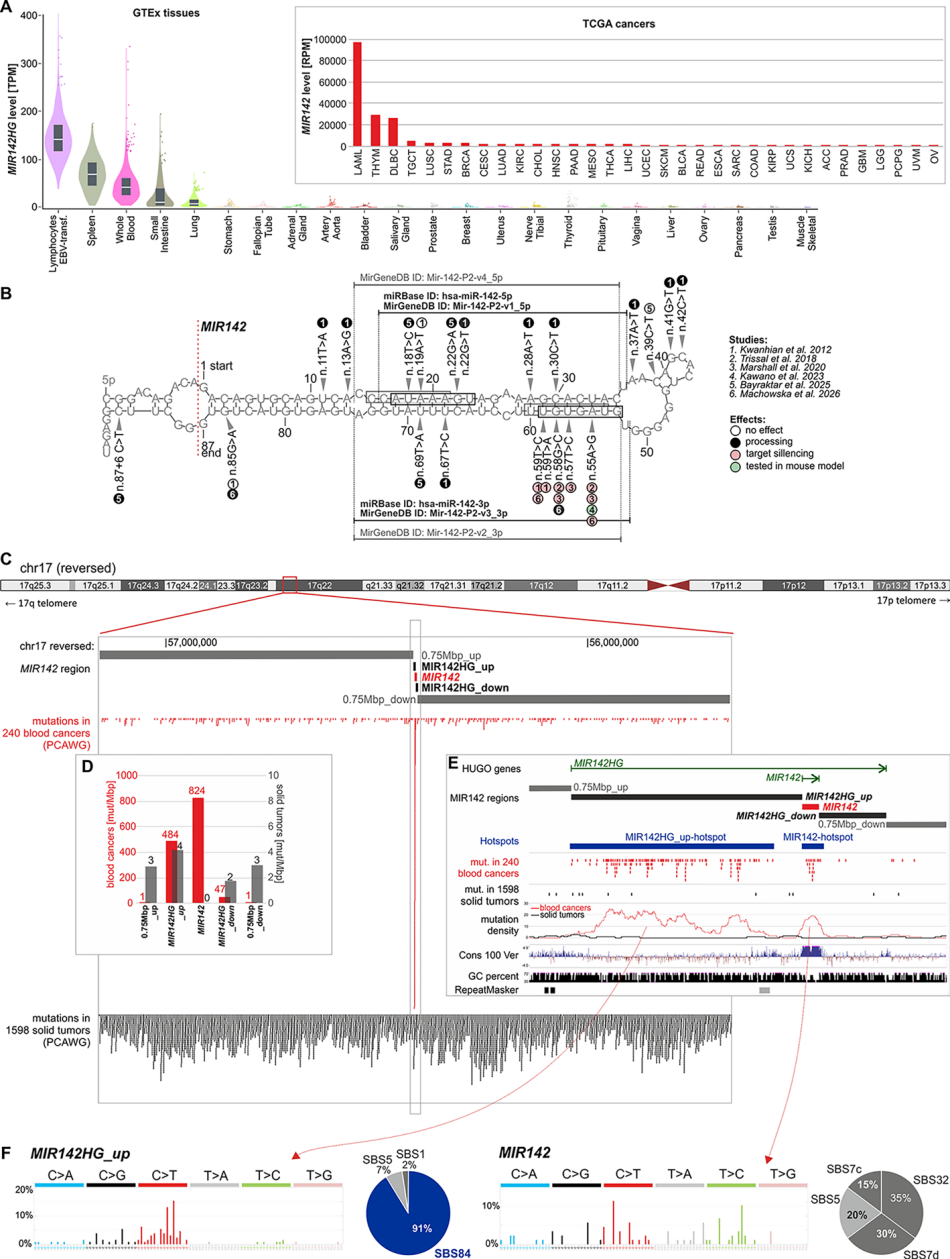
Expression and mutations of the *MIR142* locus in blood cancers.** A ***MIR142* level in cancer samples (based on TCGA data [43] via FireBrowse [https://firebrowse.org/]) and *MIR142HG* normal tissues (by GTEx [44]). Cancer type names follow TCGA project nomenclature, including LAML for AML, THYM for thymoma, and DLBC for DLBCL. **B** The structure of the miR-142 precursor, with the indicated positions of functionally tested mutations. The open circles indicate mutations with no observed effect, while the filled circles indicate mutations affecting miRNA processing (black), target repression (red), and functionally tested in a mouse model (green). Note, however, that most mutations affecting target repression were also found to alter miRNA processing, and most mutations shown to affect processing were not tested for the effect on target repression. The nucleotide positions and boundaries of *MIR142* are indicated along the precursor sequence; mature miRNAs (isomiRs) annotated in miRBase and MirGeneDB, with corresponding identifiers, are indicated above and below the precursor structure. **C** Genomic context of *MIR142* on chromosome 17 (hg19), with mapped somatic mutations identified in blood cancers and solid tumors from the PCAWG dataset. The mutation maps were visualized in the UCSC Genome Browser as “Custom tracks” displayed in a “squish” mode. The mutation lists were submitted to UCSC as separate annotation tracks in BED format with the use of the “My Data/Custom tracks” tool. Importantly, because *MIR142* is encoded on the “−” chromosome strand, the genomic orientation of the map is reversed. **D** Graph showing mutation density represented as a number of mutations per million bp [mut/Mbp] in different analyzed regions around *MIR142*. Owing to large differences, results for blood cancers (red) are plotted on the left axis, while results for solid tumors (grey) are plotted on the right axis. **E** Zoomed view of the *MIR142HG* region, with indicated mutations and hotspot regions in blood cancer and solid tumors, mutation density plot (100 bp sliding window), and mutation hotspot ranges, as well as the visualization of sequence conservation — Cons 100 Ver and GC content, and the distribution of repetitive elements (RepeatMasker) UCSC tracks. **F** Mutational signature graphs showing proportion of all single base substitutions (SBS) types (in ±1 nucleotide context) detected in *miR142HG_up* (PCAWG) and *MIR142* (mutations identified in this study combined with mutations reported previously) hotspots. The pie charts display the proportion of substitutions attributed to established mutational signatures; signature SBS84, associated with AID activity, is highlighted in blue. All figures were created using CorelDRAW Graphics Suite 2020, with graphs imported from Microsoft Excel, RNA secondary structures modeled via the mfold web server [45], and signature analysis data generated with SigProfilerAssignment.

miRNAs are short, noncoding RNA molecules that posttranscriptionally regulate the expression of many genes. They play an important role in cancer, where many miRNAs, either upregulated or downregulated, have been identified as regulators of important cancer-related processes. Still, however, very little is known about cancer somatic mutations in miRNA genes. Recently, utilizing a large cancer oncogenomic dataset generated by The Cancer Genome Atlas (TCGA; comprising over 10,000 cancer samples from more than 30 cancer types), as well as our own results of whole miRNome sequencing (WMS), we performed a comprehensive whole-genome analysis of somatic mutations in miRNA genes, defined as ~ 100-nt-long sequences encoding hairpin-shaped miRNA precursors, in cancer samples [1, 2]. Altogether, we detected over 10,000 mutations occurring in various miRNA genes, including the cancer-related miRNA genes annotated in the Cancer miRNA Census (CMC) [3]. A detailed analysis and annotation of the mutations revealed that they are distributed throughout all parts of miRNA genes, including important functional elements such as seeds, regulatory sequence/structure motifs, and DROSHA/DICER1 cleavage sites, and that they are overrepresented in certain miRNA genes. Enrichment analysis of these mutations showed that *MIR142* [annotated also as hsa-mir-142 (miRBase [4]) and Hsa-Mir-142-P2 (MirGeneDB [5])], which is specifically mutated in blood cancers, is the most frequently mutated miRNA gene across all cancer types [2]. A recurrence of *MIR142* mutations in various hematologic malignancies, particularly those of lymphoid origin, has been further validated in numerous studies, which have reported *MIR142* mutations in acute myeloid leukemia (AML), chronic lymphocytic leukemia (CLL), diffuse large B-cell lymphoma (DLBCL), follicular lymphoma (FL), and other types of B-cell non-Hodgkin lymphoma (BNHL), and have suggested these mutations as potent drivers and biomarkers of the diseases [2, 6–11]. The frequent mutations of *MIR142* in blood malignancies align with its high expression and the role it plays in different blood lineages. As shown in Fig. 1A, although *MIR142* is expressed in all cell types, it is particularly highly expressed in various blood cells, lymphoid tissues, and different blood malignancies. Importantly, *MIR142* produces mature miRNAs from both the 5p and 3p arms of its secondary precursor (pre-miR-142), respectively, miR-142-5p and miR-142-3p. However, likely owing to analytical biases, data on whether miRNAs generated from the 5p or 3p precursor arm are more prevalent remains inconsistent, even for the same cell type. Additionally, miRNA-seq data consistently show that both miR-142-5p and miR-142-3p are generated in two main versions (isomiRs) shifted at their 5′ ends (Fig. 1B). For simplicity, in this article, we will refer to one miR-142-5p and one miR-142-3p annotated in miRBase/MirGeneDB as hsa-miR-142-5p/Mir-142-P2-v1_5p and hsa-miR-142-3p/Mir-142-P2-v3_3p, respectively, which were first described and are therefore often referred to as canonical [4, 5].

## Function of *MIR142* in hematopoesis and immunity

Numerous studies, including those of *MIR142* knockout and conditional knockout mouse models, have demonstrated that *MIR142*, particularly miR-142-3p, plays a key role as a regulator of hematopoiesis, proliferation, differentiation, and maturation of various hematopoietic lineages, as well as in the regulation of innate and humoral immune responses. It was shown that animals with a deletion of *MIR142* (*MIR142*−/−), although anatomically normal, have a greatly enlarged spleen (splenomegaly) and exhibit numerous aberrations in the development of various cell lineages. Among others, *MIR142*−/− mice exhibit increased proliferation and expanded populations, as well as abnormal homeostasis of various B-cell lineages, especially spleen marginal zone B-cells, and a reduction in various T-cell lineages, particularly in the periphery [12]. *MIR142* knockout mice also exhibited a combined immunodeficiency resulting from reduced post-immunization production of antigen-specific antibodies and ineffective response to soluble and viral antigens [12]. Expression profiling of splenic B cells from the *MIR142* knockout mouse showed upregulation of many miR-142-3p (but not miR-142-5p) targets. Among the pathways with the highest number of derepressed genes were those involved in actin cytoskeleton remodeling, the generation of antigen receptor diversity through VDJ recombination, and the B-cell activating factor receptor (BAFF-R). Among other well-documented functions of *MIR142* are: regulation of cytoskeletal dynamics, control of metabolic reprogramming, and regulation of T cell homeostasis [12–14]. *MIR142* has also been implicated in autoimmunity. It has been demonstrated that downregulation of miR-142-3p, by affecting regulatory T cells (Tregs), may contribute to the development of various autoimmune diseases [15–18]. Among well-validated targets of miR-142-3p and miR-142-5p are genes playing an essential role in the biology of blood cells, including *RAC1*, a small guanosine triphosphatase (GTPase) regulating cytoskeletal dynamics and immune cell migration [10]; *ADCY9*, an enzyme catalyzing cyclic adenosine monophosphate (cAMP) production, regulating activity of regulatory Tregs, potentially contributing to autoimmunity [14]; *TGFBR1*, a key mediator of transforming growth factor (TGF)-β signaling involved in immune regulation [13]; *ASH1L* an histone lysine methyltransferase, an epigenetic regulator of HOX genes critical for hematopoietic stem cell maintenance and differentiation [19]; *IL6* a cytokine that plays a key role in immune regulation and inflammatory responses [20]; RAG2 and DNTT, playing a role in VDJ recombination and immunoglobulin diversification [12]; and *TNFRSF13C*, encoding BAFF-R, a cell surface receptor playing a role in growth, survival, and maturation of peripheral B-cells [12]. In cancer, including blood and solid tumors, *MIR142* primarily functions as a tumor suppressor and is frequently downregulated in various cancers [21–24]. The role of *MIR142* in hematopoiesis, as well as its association with various types of blood cancers, has recently been comprehensively reviewed by Huang et al. [25].

## Therapeutic potency of *MIR142*

Emerging evidence also indicates that miR-142-3p holds promise as a therapeutic agent. In 2023, Marcucci’s group reported that loss of *MIR142* is a key factor driving the progression of chronic myeloid leukemia (CML) into blast crisis, and demonstrated that systemic administration of a synthetic miR-142-3p mimic (M-miR-142) reversed leukemic stem cell metabolic reprogramming, reduced disease aggressiveness, and significantly extended survival in murine and patient-derived xenograft models [21]. Building on these findings, the authors further expanded this therapeutic approach to the immune compartment, showing that *MIR142* deficiency in T cells promotes immune escape in blast crisis CML, and demonstrated that pharmacological restoration of *MIR142* with M-miR-142 (also supplemented with a synthetic mimic of miR-142-5p) rescues T-cell metabolic fitness, effector function, and anti-leukemic activity in a mouse model [26]. This strategy was also applied to adoptive immunotherapy, where administration of M-miR-142 greatly enhanced the persistence and anti-leukemic effectiveness of IL1RAP-targeted chimeric antigen receptor T-cell (CAR-T) cells in AML mouse models, leading to decreased leukemia burden and prolonged animal survival [27]. Overall, these preclinical studies suggest that miR-142-3p/5p replacement represents a versatile therapeutic approach that can boost both endogenous and engineered T-cell-based antileukemic responses, meriting further translational research.

## Functional study of mutations in *MIR142*

The identification of recurrent mutations in *MIR142* has prompted researchers to explore their molecular and physiological impacts, including their potential to drive cancers. The locations of all functionally tested mutations described in this chapter are shown in Fig. 1B. First, Kwanhian et al. [10], using cellular models and molecular techniques such as luciferase reporter assays, western blotting, and miRNA northern blotting, examined mutations identified in DLBCL samples. They showed that two mutations at the seventh nucleotide (for simplicity, we number the seed positions relative to the seed of canonical miR-142-3p, which is marked in Fig. 1B as a rectangle with a black outline) of the miR-142-3p seed, n.59 T > C and n.59 T > A, affect the silencing of previously validated miR-142-3p targets such as *RAC1* and *ADCY9*. They also found that one of these mutations (n.59 T > C) results in molecular reprogramming and the recognition of novel mutation-specific targets in the 3′-untranslated region (UTR) of *ZEB2*, which wild-type miR-142-3p does not recognize. They then showed that mutated versions of miR-142-3p effectively reduce *ZEB2* transcript and protein levels, suggesting a gain-of-function effect of the mutation. ZEB2 is a transcription regulator involved in cancer by controlling E-cadherin and the epithelial-to-mesenchymal transition. Subsequently, Kwanhian et al. revealed that mutations in other parts of the miRNA precursor might also impact *MIR142* function by altering the efficiency and accuracy of miRNA biogenesis [10].

Next, Trissal et al. analyzed n.55A > G and n.58G > C mutations, located at the third and sixth positions of the miR-142-3p seed in the context of leukemic transformation and AML development [19]. Using miRNA expression vectors, polymerase chain reaction (PCR)-based quantification, and luciferase reporter assays, they first demonstrated that these mutations affect the levels and ratios of miR-142-5p and miR-142-3p, and then showed that miRNAs from the mutated *MIR142* fail to repress canonical targets of miR-142-3p, *TGFBR1*, and *RAC1*. The authors further showed that, in *MIR142* knockout mice, loss of *MIR142* affects the differentiation of hematopoietic stem/progenitor cells, reducing their erythroid and lymphoid potential while enhancing their myeloid potential, thereby promoting leukemic transformation. Mechanistically, it was proposed that the functional loss of *MIR142* leads to derepression of the histone methyltransferase *ASH1L* (a target of both miR-142-5p and miR-142-3p), which positively regulates *HOHA9*/*A10*, *HOXA* family genes involved in the self-renewal of hematopoietic progenitors. Sustained expression of *HOXA9/A10* in bone marrow myeloid progenitors induces a myeloproliferative phenotype, contributing to leukemic transformation. Trissal et al. also suggested that *MIR142* mutations work in parallel and enhance the leukemic effects of *IDH2* oncogenic mutations [19].

The deleterious impact of three miR-142-3p seed mutations (n.55A > G and n.58G > C, previously analyzed by Trissal et al., and n.57 T > C located at the 5th nucleotide of the seed) on the silencing of miR-142-3p targets, as well as their synergy with the *IDH2* hotspot mutation (R140Q), were confirmed and further explored by Marshall et al. [28]. Using a mouse model, they showed that leukemia developed only in recipients of the double mutations, and neither *MIR142* loss nor *IDH2* mutation alone was sufficient to induce leukemia. This study further supported the miR-142-ASH1L-HOXA axis, demonstrating that shRNA-mediated knockdown of *ASH1L* partially suppressed abnormal myeloid expansion in double-mutant cells.

The clustered regularly interspaced short palindromic repeats (CRISPR)/Cas9-generated knock-in mouse model with the miR-142-3p seed n.55A > G mutation showed a decreased birth rate and rapid mortality in mice homozygous for the mutation [29]. The study also found that mice with the mutation mirror the overall phenotype of *MIR142* knockout mice [12], including splenomegaly and abnormal counts and development of various blood cell types [29]. Transplanting bone marrow from a mouse heterozygous or homozygous for the n.55A > G mutation into a lethally irradiated recipient mouse caused an expansion of CD4^+^ and CD8^+^ T-cell lineages in peripheral blood, bone marrow, spleen, and thymus, while reducing B cells in peripheral blood, and led to the development of CD8^+^ T cell leukemia. To explain the observed effects and connect the mutation with expression changes, the authors performed RNA-seq analysis comparing wild-type T-cells with pre-leukemic and leukemic CD8^+^ T-cells derived from marrow heterozygous for the n.55A > G mutation. The analysis showed a substantial increase in the expression of genes involved in cancer proliferation, including those from the Myc and mTORC1 pathways, and a decrease in genes involved in immune functions and apoptosis. Further analysis of genes differentially expressed in mutant leukemic T cells revealed upregulation of the direct targets of wild-type miR-142-3p and a decrease in targets specific for mutant miR-142-3p, indicating both loss- and gain-of-function effects of the mutation [29].

Recently, Calin’s group identified several somatic mutations in *MIR142* in patients with CLL [30]. These mutations were located throughout different regions of the miRNA gene/precursor. Using cellular models transfected with plasmids containing both wild-type and mutant *MIR142*, the researchers examined how five of these mutations affected primary and secondary miR-142 precursors, as well as mature miR-142-5p and miR-142-3p levels. They found that, although these mutations had little or no effect on precursor levels, four significantly decreased miR-142-3p, with two also reducing miR-142-5p levels. One mutation, n.87 + 6C > T, located in the 3p flanking region, which had the strongest impact on both miR-142-3p and miR-142-5p, disrupted the conserved CNNC motif involved in miRNA biogenesis [30]. The authors showed that this mutation impairs the recruitment and interaction of the precursor with serine/arginine-rich splicing factor 3 (SRSF3), important for DROSHA-mediated processing of the primary transcript. In addition to affecting conserved regulatory motifs, the effects of miRNA gene mutations outside seed regions may also depend on their impact on miRNA precursor structure and on modifications to miRNA duplex thermodynamic properties. This way, mutations in the 5p arm can affect the function/level of miRNA-3p and vice versa. A more detailed discussion of the mechanisms by which mutations impact miRNA processing was presented recently by Machowska et al. [31].

Lastly, using miRNA-seq data from thousands of cancer samples within The Cancer Genome Atlas (TCGA), we compared miRNA levels and structures in samples with mutations in miRNA genes [32]. This approach allowed, for the first time, the study of mutation effects in their natural genomic context within actual samples where mutations were detected, thereby avoiding the potential biases inherent in artificial models. Our analysis revealed that four mutations (n.55A > G, n.58G > C, n.59 T > C in the canonical miR-142-3p seed, and n.85G > A in the 3′ flanking region), found in AML or DLBCL samples, altered the distribution of the two main miR-142-3p isomiRs. Despite different locations, all mutations increased the proportion of the canonical miR-142-3p at the expense of the isomiR extended by one nucleotide at the miRNA 5’-end, which was dominant in wild-type samples. We confirmed the effects of three mutations (n.55A > G, n.59 T > C, and n.85G > A) on isomiR profiles experimentally, using artificially generated expression vectors, and showed with luciferase assays that two mutations (n.55A > G, n.59 T > C) impair miR-142-3p’s ability to repress its three selected, well-validated targets (*TGFBR1*, *RAC1*, and *ASH1L*). Because most functional analyses of *MIR142* mutations have mainly focused on seed mutations so far, further research is needed to thoroughly examine mutations in other regions of *MIR142*, including sequences that encode the terminal loop and flanking regions of the miR-142 precursor.

## Genomic context and profile of *MIR142* mutations

To date, all studies of *MIR142* mutations have been limited to a relatively narrow ~ 100-bp-long sequence of *MIR142*, spanning only the hairpin-shaped part of miR-142 precursor. There is, however, no information on how far the mutated region extends and whether mutations also occur in other parts or regulatory elements of the miR-142 transcriptional unit, also known as the *MIR142* host gene (*MIR142HG;* ~ 1.6 kb). Therefore, to determine the extent of the mutated region, its structure, and how it diverges from the regional background mutation rate, we analyzed mutations not only in *MIR142* but also in the wider genomic context, including *MIR142HG* [~ 1.2 kb upstream (*MIR142HG_up*) and ~ 0.4 kb downstream (*MIR142HG_down*) of *MIR142*], and 1.5 Mb genomic region surrounding *MIR142* (0.75 Mbp_up and 0.75 Mbp_down) (Fig. 1C). For the analysis, we used whole-genome sequencing data of 1838 cancer samples from the Pan-Cancer Analysis of Whole Genomes (PCAWG) project [33], including 240 blood cancer samples, such as BNHL (*n* = 98; predominantly DLBCL and FL), CLL (*n* = 95), myeloid neoplasms (*n* = 20; predominantly AMLs), and myeloproliferative neoplasms (MPN) (*n* = 25) samples. As shown in Fig. 1C, mutations in blood cancer samples are strongly enriched at the *MIR142* locus, forming a hotspot that is clearly distinct from the genomic background. The formal analysis of mutation density revealed that mutation occurrence is highest in *MIR142*, moderately elevated in *MIR142HG*, particularly in *MIR142HG_up*, and very low (accidental) in flanking genomic regions (Fig. 1D). The hotspot is not present in solid tumors, where there are barely any mutations in the locus. A closer examination of the hotspot revealed that it consists of two distinct, densely mutated regions (Fig. 1E). A narrow (~ 80 bp) sharp peak of mutation density coinciding with the highly conserved region containing *MIR142*, and a wider (~ 0.9 kb) region located upstream of *MIR142*, extending from the *MIR142HG* promoter almost the entire length of *MIR142HG_up* (the ranges of the hotspots are shown in Fig. 1E). We validated a similar pattern of mutation distribution in an independent dataset of ~ 5000 metastatic cancer samples from the Hartwig Medical Foundation (HMF) [34]. An increased density of mutations at the *MIR142* locus was observed in HMF samples, despite the dataset including only a small number of lymphoid malignancies, specifically 25 BNHL samples (Supplementary Fig. S1). The average variant allele frequency (VAF) of PCAWG and HMF mutations detected in *MIR142* was 27%, with a range of 8–55%. We also observed that mutations in the *MIR142* hotspot tend to occur more frequently in females than in males, affecting 12% of female and 5% of male BNHL samples (Fisher’s exact test, *p* = 0.047).

**Fig. 2:**
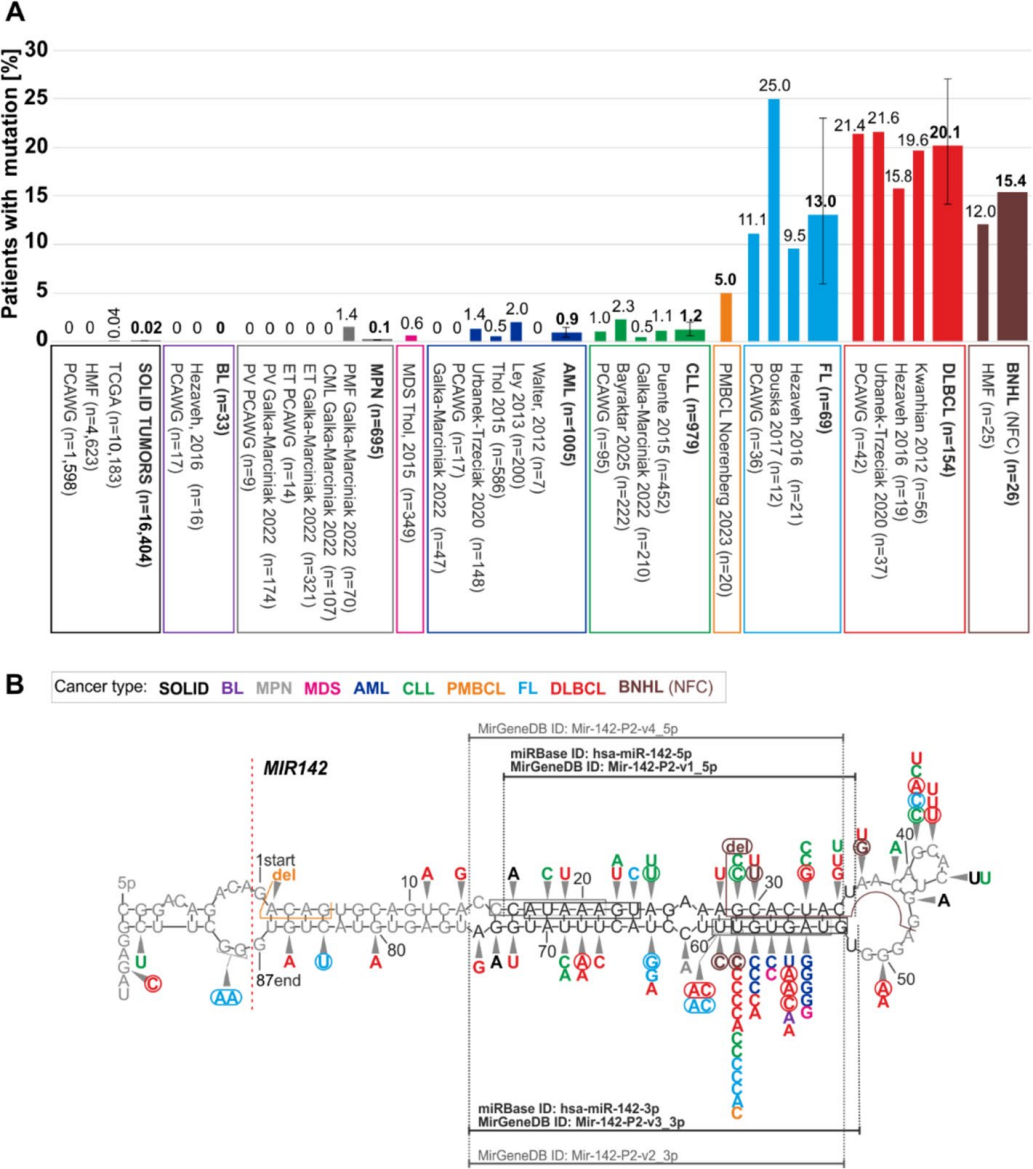
Distribution of *MIR142* mutations.** A** Frequency of samples with *MIR142* mutations in different cancer types; narrow bars indicate results of individual studies, and wide bars indicate results of integrative analysis combining mutations from all sources; error bars indicate 95% confidence intervals. *BL* Burkitt lymphoma, *MPN* myeloproliferative neoplasms, including *PV *polycythemia vera, *ET *essential thrombocythemia, *CML* chronic myeloid leukemia, *PMF* primary myelofibrosis, *MDS* myelodysplastic syndrome, *AML* acute myeloid leukemia; *CLL* chronic lymphocytic leukemia, *PMBCL* primary mediastinal B-cell lymphoma, *FL* follicular lymphoma, *DLBCL* diffuse large B-cell lymphoma, and *BNHL (NFC)* B-cell non-Hodgkin lymphoma (non-further classified). **B** Distribution of *MIR142* mutations mapped to the secondary structure of the miR-142 precursor, with colors indicating tumor types (as in **A**), and circled identifiers marking mutations identified in this study (PCAWG and HMF). Mutations were annotated using the miRMut protocol [46], according to HGVS nomenclature (https://hgvs-nomenclature.org), with reference to the *MIR142* sequence as annotated by the HUGO Gene Nomenclature Committee (www.genenames.org). The gene nucleotide numbering, along with the first and last positions of the gene, are shown adjacent to the sequence. The mature miRNA (isomiR) sequences, with miRBase and MirGeneDB identifiers, are indicated above and below the miRNA precursor structure, with corresponding seed sequences boxed by rectangles. Mature miRNA strands are marked in bold, and seed regions are highlighted in gray.

Given prior suggestions that mutations in *MIR142* may be an effect of accidental off-target activity of activation-induced cytidine deaminase (AID) [9, 35], which mediates somatic hypermutation and class-switch recombination of immunoglobulins in B cells [36], we sought to investigate whether these mutations exhibit features characteristic of AID-associated mutagenesis. For this purpose, we used SigProfiler from COSMIC [37] to analyze the mutational signatures of hotspot mutations, separately for the *MIR142* and *MIR142HG_up* hotspots (Fig. 1F). Owing to the limited number of mutations identified in the *MIR142* hotspot in PCAWG (*n* = 19) and HMF (*n* = 4) (Fig. 2A; Supplementary Table S1), we supplemented the set of *MIR142* mutations with 71 mutations reported previously in different studies [6–11, 30, 38, 39], including mutations detected in blood cancers in the TCGA dataset [2] (Supplementary Table S1). As shown in Fig. 1F, the analysis revealed a markedly different mutation pattern in *MIR142* and *MIR142HG_up* hotspots. Notably, 91% of the mutations in the *MIR142HG_up* hotspot were associated with the SBS84 signature, which is attributed to AID activity. Conversely, mutations in *MIR142* (both identified in this study (*n* = 23) and including the mutations identified previously (*n* = 94)) did not exhibit such an evident association with AID-related signatures. Also, the frequency of C > T and G > A substitutions, which may result directly from AID cytidine deaminase activity, was much lower in *MIR142* (28%) than in the *MIR142HG* hotspot (71%; Fisher’s exact test, *p* = 7 × 10⁻^10^). It is worth noting, however, that owing to the heterogeneous origin and the low number of mutations, the analysis of *MIR142* mutations had limited power, so the possibility that individual mutations are AID-related cannot be ruled out. The mutation patterns may also be influenced by GC content, which is slightly lower in *MIR142*, reducing the potential for AID-induced C > T transitions. However, consistent with our results, Rheinbay et al. reported that seven out of eight analyzed mutations within the mature miR-142-3p sequence were not attributed to AID [40]. The mutations in *MIR142* were predominantly (35%) associated with the SBS32 signature, occurring in lymphomas and related to immunosuppressive treatment with azathioprine. Additionally, while the occurrence of mutations in *MIR142* and *MIR142HG_up* is closely associated, many samples with *MIR142* mutations do not show mutations in *MIR142HG_up* (8 out of 19), and vice versa (41 out of 52).

## Occurrence of *MIR142* mutations in different blood cancers

Mutations identified in *MIR142* (PCAWG + HMF) were detected in 21% of DLBCL samples, 11% of FL samples, 1% of CLL samples, and 12% of non-further classified (NFC) BNHL samples. No mutations were detected in small cohorts of AML and Burkitt lymphoma (BL). Additionally, no mutations were detected in thousands of solid tumors (Fig. 2A). To more precisely determine the frequency of *MIR142* mutations in the analyzed hematologic cancers, we combined the mutations identified in this study with those reported previously [2, 6, 30, 38, 41], considerably increasing the size of the analyzed cohorts. As shown in Fig. 2A, despite some discrepancies, primarily due to the small sample sizes of certain studies, the collected data generally demonstrated high concordance among the studies. The integrative analysis showed that *MIR142* mutations occur in 20.1% [95% confidence interval (CI) 14–27%] of DLBCL samples, 13% (6–23%) of FL samples, 1.2% (0.6–2%) of CLL samples, 0.9% (0.4–1.8%) of AML samples, and in no sample of BL (the only mutation in BL was identified in the Raji cell line [2]); only few incidental mutations occurred in solid tumors (0.02%, CI 0.01–0.06%). The analysis also showed that, although DLBCL and FL are the most frequently mutated, they are still substantially understudied, resulting in relatively high CIs. However, it should be noted that differences in mutation occurrence between cancer subtypes may constitute another source of variation. The fact that no *MIR142* mutations were detected in primary BL samples, a malignancy characterized by high proliferative activity and extensive AID-associated mutagenesis (with ~ 27% of mutations attributed to AID [42]), further supports the notion that *MIR142* mutations are independent of AID-mediated mutagenesis. It also reflects well-known differences in lymphomagenesis of DLBCL and FL, characterized by a much higher complexity and mutation load, compared with the genetically simpler, MYC-addicted pathogenesis of BL.

## Distribution of mutations within *MIR142*

As shown in Fig. 2B, although the mutations are distributed along almost the entire *MIR142*, they cluster especially around the seed of miR-142-3p, and their distribution differs markedly among different cancer types. All AML and MDS mutations are located in the miR-142-3p seed (binomial distribution, *p* = 3.8 × 10^−11^ and 4.1 × 10^−3^, respectively), DLBCL mutations occur in different parts of the precursor but also are enriched in the miR-142-3p seed (*p* = 1.3 × 10^−5^), FL mutations tend to be more prevalent in the 3p arm of the precursor, including 3p seed, whereas CLL mutations tend to be more prevalent in the 5p arm of the precursor. Notably, since MDS often progresses into and serves as a precursor to AML, the occurrence of similar mutations in AML and MDS may indicate that at least some of the *MIR142* mutations occur early in disease development. It is also worth noting that certain positions in the precursor are mutated much more frequently than others, creating hotspots within the *MIR142* hotspot. The most frequently mutated position (*n* = 15) is n.59T, located in the seventh nucleotide of the miR-142-3p seed, substituted with either C or A. Other hotspot positions, mutated five or more times, are n.41G (in the terminal loop), n.55A, n.56G, and n.58G.

## Conclusions

Here, we characterized the genomic context of *MIR142* mutations in hematologic malignancies and demonstrated that mutations in the *MIR142* gene in hematological malignancies create a clearly distinct hotspot, not extending beyond the gene boundaries, and concentrated mainly within the sequence of pre-miR-142. We also demonstrated that the *MIR142* hotspot is closely located to another hotspot (*MIR142HG_up*), which exhibits distinct characteristics, and that both hotspots are clearly distinct from the regional mutation rate. We documented differences in the frequency of *MIR142* mutations across hematologic malignancies and showed that the distribution of mutations within *MIR142* varies substantially by tumor type. These findings indicate that *MIR142* alterations are not a byproduct of an elevated mutational load but most likely are subject to positive selection, highlighting their potential biological and clinical relevance, which warrants further investigation, including validation in larger cohorts of specific hematologic malignancies, functional characterization, and verification of whether these mutations arise during early B-cell clonal expansions or reflect later selection. Additionally, this review highlights the crucial role of *MIR142* in regulating hematopoiesis and the function of various hematologic cell lineages, as well as different aspects of immunity. It suggests that, whether or not *MIR142* mutations are drivers of cancer development, they may affect the phenotype of patients with certain blood cancers. Although further studies in larger cohorts of specific cancer types and association studies of mutations with clinical phenotypes are still needed, given *MIR142*’s role in hematopoiesis and the fact that the occurrence of *MIR142* mutations is not random and may be associated with a specific molecular pattern of cancers, affecting particular signaling and metabolic pathways, and potentially defining a specific subtype of the disease, a far-reaching goal may be the utilization of *MIR142* mutations as biomarkers of functional *MIR142* deficiency or to help distinguish certain subtypes of hematologic malignancies. Furthermore, as shown, *MIR142* deficiency contributes to the development of blood cancers; supplementing miR-142, for example, with miRNA mimics, could potentially be used as a treatment for blood cancers, as already demonstrated in the CML mouse model.

## Supplementary Information

Additional file 1.

Additional file 2.

## Data Availability

The study was based on whole-exome sequencing data from the publicly available PCAWG dataset generated by the International Cancer Genome Consortium (MAF file, version from 25 November 2019; PCAWG-ICGC dataset), and the dataset from the Hartwig Medical Foundation collection of metastatic cancer samples (accession no. DR-275).

## Abbreviations

AID: Activation-induced cytidine deaminase
AML: Acute myeloid leukemia
BNHL: B-cell non-Hodgkin lymphoma
CLL: Chronic lymphocytic leukemia
CML: Chronic myeloid leukemia
DLBCL: Diffuse large B-cell lymphoma
ET: Essential thrombocythemia
FL: Follicular lymphoma
HMF: Hartwig Medical Foundation
NFC: Non-further classified
MDS: Myelodysplastic syndrome
*MIR142HG_up*: ~ 1.2 kb upstream of *MIR142* gene
*MIR142HG_down*: ~ 0.4 kb downstream of *MIR142* gene
MPN: Myeloproliferative neoplasms
PBMCL: Primary mediastinal B-cell lymphoma
PCAWG: Pan-Cancer Analysis of Whole Genomes project
PMF: Primary myelofibrosis
PV: Polycythemia vera
