# Supplementary material for: Genomic context of mutations in MIR142 in blood cancers: summary and integrative analysis
Source: Cell Mol Biol Lett. 2026 May 18;31:127. doi: 10.1186/s11658-026-00933-9 (PMC13435480; doi:10.1186/s11658-026-00933-9)
Supplement: Supplementary file 1 [file 11658_2026_933_MOESM1_ESM.pdf]

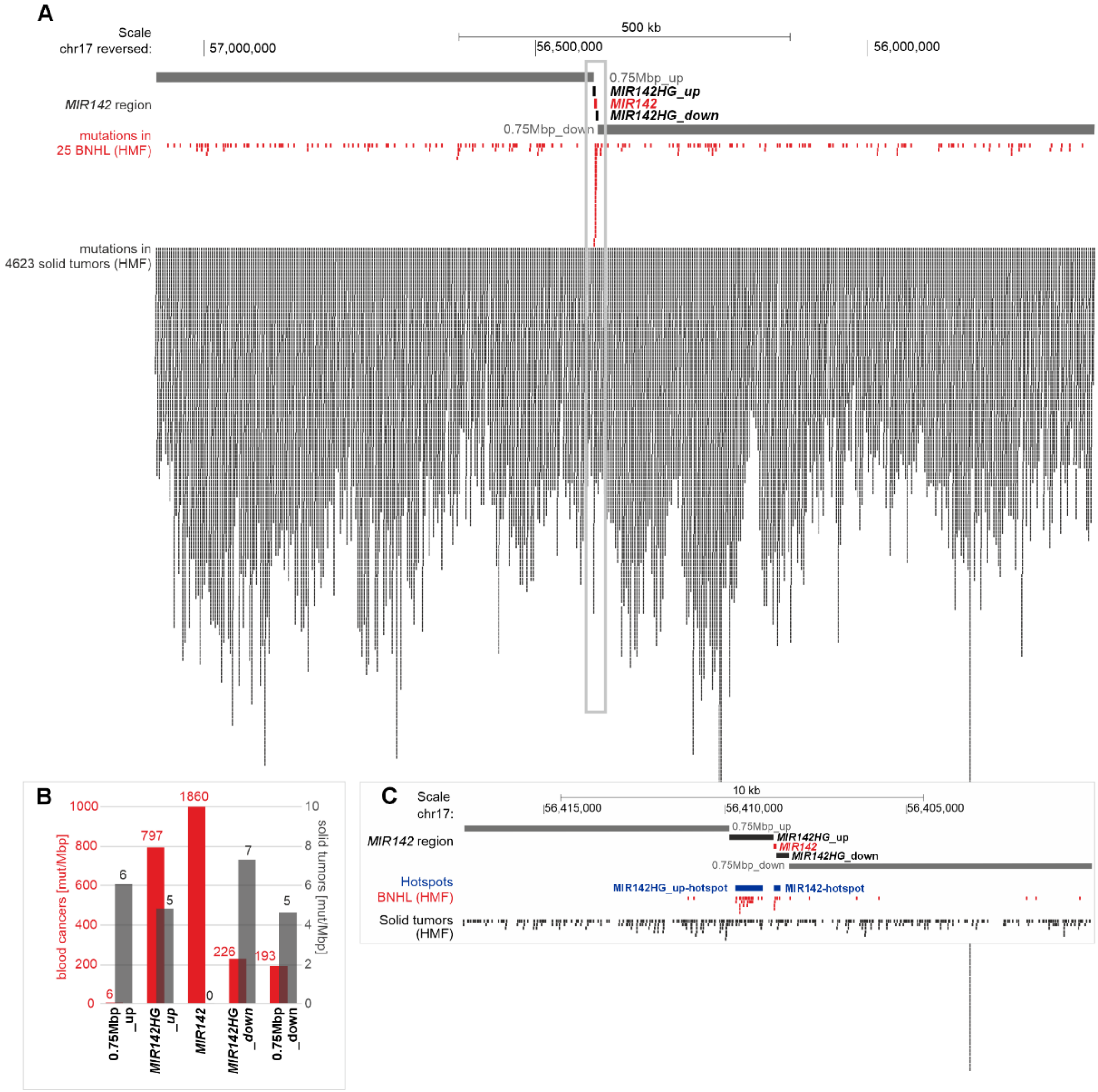


**Supplementary Figure S1. A)** Map of somatic mutations identified in HMF-deposited blood cancer (BNHLs, N=25) and solid tumor (N=4,623) samples (visualized with the UCSC Genome Browser). **B)** Graph showing mutation density. **C)** Zoomed-in view of the MIR142HG region. The overall figure schemes are as in Figures 1C, 1D, and 1E, respectively.
